# Ocular manifestations in Iranian patients referred to rheumatology clinics from 2018 to 2020

**DOI:** 10.1002/iid3.863

**Published:** 2023-05-08

**Authors:** Meharan Pournazari, Tara Hashemi, Mahsa Zarpoosh, Parsa Amirian

**Affiliations:** ^1^ Department of Rheumatology, Imam Reza Hospital Kermanshah University of Medical Sciences (KUMS) Kermanshah Iran; ^2^ Kermanshah University of Medical Science (KUMS) Kermanshah Iran

**Keywords:** anterior uveitis, Behçet's disease, blurred vision, eye diseases, ocular manifestation, rheumatology diseases

## Abstract

**Aim:**

Autoimmune diseases are presented with many signs and symptoms. Eyes are commonly involved in these diseases. This study aimed to estimate the prevalence of different ophthalmological complications in patients with and without immune‐mediated rheumatological diseases.

**Methods:**

Patients who were referred to Kermanshah's rheumatologic clinics by an ophthalmologist from 2018 to 2020 for a rheumatologist visit were included. A checklist for extracting data from medical files; containing symptoms, organ involvement, ocular diseases diagnosed by an ophthalmologist, rheumatologic diseases diagnosed by a rheumatologist, lab tests, and disease progression was created. After we evaluated the medical data, we found that 54 patients out of 106 were diagnosed to have immune‐mediated rheumatological diseases. Patients were divided into two groups; the first group included patients with diagnosed immune‐mediated rheumatologic disease and ophthalmic complications; patients with no known immune‐mediated rheumatological disease were considered the second group. The obtained information was analyzed using statistical tests.

**Results:**

One hundred and six patients participated in this study, 67% of whom were females. The most common ocular symptom was blurred vision (49%). Involvement of both eyes (43.4%) was more common than single left or right eye involvement. The most common ophthalmic disease was anterior uveitis (35.8%). The most common rheumatologic disease was Behçet's disease (21.7%). Hypertension and hypothyroidism were the most common comorbidities; 36.7% of the patients had skin and mucous involvement, and 37.7% had joint involvement. In follow‐up of the ophthalmic symptoms, most patients were controlled partially. Ophthalmic diseases, laboratory tests, joint involvement, skin and mucous involvement, and lung involvement were associated with rheumatologic diseases.

**Conclusion:**

Early diagnosis of ocular involvement in rheumatologic diseases is crucial to prevent adverse complications. The results can be beneficial for a better perception of ophthalmic symptoms and diseases among patients with autoimmune diseases.

## Graphical abstract

Investigating the frequency and outcome of ocular diseases among Iranian patients with autoimmune diseases is barely studied; to determine the most common ophthalmic symptoms and diseases among patients with autoimmune diseases, this study was conducted.

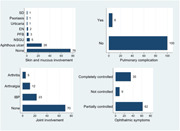

## 1 INTRODUCTION

Autoimmune diseases (ADs) affect approximately 7.6%–9.4% of the general population worldwide, based on recent studies.^1^ The prevalence of various rheumatologic diseases among Iranian patients are as follows: Rheumatoid arthritis (RA) 0.37%, Seronegative spondyloarthritis 0.24%, Ankylosing spondylitis (AS) 0.12%, Systemic lupus erythematosus (SLE) 0.06%, Behçet's disease (BD) 0.08%.^2^

ADs can show various signs and symptoms. The eyes are frequently involved in these diseases, and ocular manifestations can be the early presentation of rheumatologic diseases.^3^ Ocular complications can have inflammatory, vascular, infectious, or iatrogenic causes, with symptoms ranging from eye dryness to blindness.^4^, ^5^ The most common ocular involvement in AD patients consists of keratoconjunctivitis sicca, episcleritis, scleritis, uveitis, vitritis, retinal vasculitis, and panophthalmitis.^6^

Uveitis is one of the major causes of visual loss and blindness, and based on the primarily affected site; it is categorized into anterior, intermediate, posterior, and panuveitis.^7^, ^8^

Uveitides associated with juvenile idiopathic arthritis (JIA) are severe conditions with different visual outcomes; uveitis is also one of the most common eye complications in seronegative spondyloarthropathies.^8^, ^9^, ^10^ Acute anterior uveitis is the most common extra‐articular presentation of AS and occurs in almost 40% of patients; it can also be observed as an early sign before diagnosing AS.^6^ Ocular complications, including uveitis, can occur without rheumatologic implications too. Previous studies have shown that 30% to 60% of uveitis cases are idiopathic.^11^

The eyes are the most commonly affected organ in BD. The most common forms of involvement are retinal vasculitis and panuveitis; the most horrifying ocular complication of BD is double‐sided scarring panuveitis since it might rapidly lead to permanent blindness.^12^, ^13^ Other rheumatologic diseases, namely sarcoidosis, vasculitides, and other spondyloarthropathies, can also demonstrate some forms of eye involvement well before diagnosis of the background disease.^6^

To estimate the prevalence of different ophthalmological complications in patients diagnosed with rheumatologic diseases and suspected patients with ocular complications, we separated them into two groups (rheumatologic group and control group); we compared these two types of patients to have a better understanding of ocular complications in immune‐mediated rheumatological diseases patients; and suspected AD patients.

## 2 MATERIALS AND METHODS

### 2.1 Study population

The current study is a retrospective cross‐sectional study of patients referred to rheumatology clinics by an ophthalmologist in Kermanshah, Iran, from 2018 to 2020. We collected data retrospectively through the medical files; 106 patients were comprised, after an initial physical examination and requesting appropriate imaging and laboratory tests by a rheumatologist for each patient, seven different immune‐mediated rheumatological diseases based on the College of Rheumatology (ACR) classification criteria of rheumatologic diseases were diagnosed in 54 patients (Tables 2 and 3), and 52 patients did not satisfy any ACR classification criteria for immune‐mediated rheumatological diseases.

We divided patients into two groups, patients who met the ACR classification criteria of rheumatologic diseases were designated as the first group (rheumatologic group), and patients with no diagnosed rheumatologic diseases according to the ACR classification criteria were considered as the second group (control group).

### 2.2 Data collection

By using a dedicated checklist following information, including symptoms, different organ involvements, ocular disease diagnosed by an ophthalmologist, rheumatologic disease diagnosed by a rheumatologist, lab tests, and disease progression was recorded, then a database in Microsoft Excel Microsoft (Microsoft Corp.) was created.

### 2.3 Statistical analysis

The database was then transformed to STATA 17 (StataCorp. 2021. Stata Statistical Software: Release 17: StataCorp LLC.) statistical software, and variables, namely: sex, age, type of rheumatologic disease, type of ocular involvement, ophthalmic symptoms, the affected eye, comorbidity were analyzed. The results were collected as means, standard deviation, the percentage for continuous variables, and frequency distribution for categorical variables. To prove an association between variables, we used the Chi‐square independence test was used, and then we drew charts and graphs.

### 2.4 Ethics approval

This study was performed in line with the principles of the Declaration of Helsinki. Approval was granted by the Ethics Committee of Kermanshah University of Medical Sciences (IR.KUMS.REC.1400.430).

## 3 RESULTS

One hundred and six patients were enlisted in the study, 71 of whom were females, and 35 were males. The minimum age was two, and the maximum was 83 years, with a mean age of 40.43 ± 13.26 years. Table 1 shows age and sex distribution.

**Table 1:** Distribution of sex and age groups.

Age group (year)	Sex				Total	%
	No. of female	%	No. of male	%		
≤9	1	0.94	0	0.00	1	0.94
10–24	7	6.60	0	0.00	7	6.60
25–39	33	31.13	14	13.21	47	44.34
40–54	24	22.64	16	15.09	40	37.74
55–69	6	5.66	3	2.83	9	8.49
≥70	0	0.00	2	1.89	2	1.89
Total	71	66.98	35	33.02	106	100.00

Ocular symptoms affecting both eyes simultaneously were more common (43.40%), and one‐sided involvement of the left or right eye was almost the same (left eye 30.19% and right eye 26.42%). Anterior uveitis was the most prevalent ophthalmic disease in both groups, and BD was the most frequently diagnosed rheumatologic disease.

Approximately half of the patients suffered from blurred vision; ophthalmic disease diagnoses and ophthalmic symptoms in the rheumatologic and control groups are listed in Tables 2 and 3, respectively. Comorbidities were assessed as well, and 20 comorbidities were found among 16 patients (four patients had two comorbidities), and 90 patients had no significant comorbidity. Hypertension and hypothyroidism were the most common ones, with seven cases each, followed by three diabetes mellitus type 2 (DM2), two chronic kidney disease (CKD), and one hepatitis (silent hepatitis B).

**Table 2:** Ophthalmological diagnoses for each rheumatologic disease and control group.

Ophthalmic disease	Rheumatologic disease							Control	Total
	AS	BD	VKH	JRA	OS	Sarcoidosis	RV		
Anterior uveitis	13 (12.2%)	2 (1.8%)	‐	1 (0.9%)	3 (2.8%)	1 (0.9%)	‐	18 (16.9%)	38 (35.8%)
Anterior uveitis & scleritis	2 (1.8%)	‐	‐	‐	‐	‐	‐	0 (0.0%)	2 (1.8%)
Anterior uveitis & episcleritis	‐	1 (0.9%)	‐	‐	‐	‐	‐	1 (0.9%)	2 (1.8%)
Anterior uveitis & retinitis	‐	1 (0.9%)	‐	‐	‐	‐	‐	0 (0.0%)	1 (0.9%)
Anterior uveitis & posterior uveitis	‐	‐	‐	‐	‐	‐	‐	1 (0.9%)	1 (0.9%)
Episcleritis	‐	1 (0.9%)	‐	‐	‐	‐	‐	3 (2.8%)	4 (3.7%)
Intermediate uveitis	‐	1 (0.9%)	‐	‐	‐	‐	‐	8 (7.5%)	9 (8.4%)
Intermediate uveitis & Episcleritis	‐	1 (0.9%)	‐	‐	‐	‐	‐	0 (0.0%)	1 (0.9%)
Pan Ophthalmitis	‐	2 (1.8%)	‐	‐	‐	‐	‐	1 (0.9%)	3 (2.8%)
Pan uveitis	3 (2.8%)	7 (6.6%)	‐	‐	‐	1 (0.9%)	1 (0.9%)	7 (6.6%)	19 (17.9%)
Posterior uveitis	1 (0.9%)	6 (5.6%)	1 (0.9%)	‐	‐	3 (2.8%)	‐	11 (10.3%)	22 (20.7%)
Posterior uveitis & Retinitis	‐	1 (0.9%)	‐	‐	‐	‐	‐	1 (0.9%)	2 (1.8%)
Retinitis	‐	‐	1 (0.9%)	‐	‐	‐	‐	0 (0.0%)	1 (0.9%)
Scleritis	‐	‐	‐	‐	‐	‐	‐	1 (0.9%)	1 (0.9%)
Total	19 (17.9%)	23 (21.7%)	2 (1.8%)	1 (0.9%)	3 (2.8%)	5 (4.7%)	1 (0.9%)	52 (49.0%)	106 ≈ (100%)

Abbreviations: AS, ankylosing spondylitis; BD, Behçet's disease; JRA, juvenile rheumatoid arthritis, OS, other spondylopathies; RV, retinal vasculitis; VKH, Vogt–Koyanagi–Harada disease.

**Table 3:** Ophthalmic symptoms in each rheumatologic disease and control group.

Ophthalmic symptoms	Rheumatologic disease							Control	Total
	AS	BD	VKH	JRA	OS	Sarcoidosis	RV		
Blurred vision	6 (5.6%)	14 (13.2%)	1 (0.9%)	1 (0.9%)	3 (2.8%)	3 (2.8%)	‐	24 (22.6%)	52 (49.0%)
Blurred vision & redness	3 (2.8%)	5 (4.7%)	‐	‐	‐	1 (0.9%)	‐	11 (10.3%)	20 (18.8%)
Blurred vision & redness & pain	2 (1.8%)	2 (1.8%)	‐	‐	‐	‐	‐	5 (4.7%)	9 (8.4%)
Metamorphopsia & blurred vision	‐	‐	1 (0.9%)	‐	‐	‐	‐	0 (0.0%)	1 (0.9%)
Pain	‐	1 (0.9%)	‐	‐	‐	‐	1 (0.9%)	0 (0.0%)	2 (1.8%)
Photophobia & redness	‐	1 (0.9%)	‐	‐	‐	‐	‐	0 (0.0%)	1 (0.9%)
Redness	8 (7.5%)	1 (0.9%)	‐	‐	‐	1 (0.9%)	‐	11 (10.3%)	21 (19.8%)
Total	19 (17.9%)	23 (21.7%)	2 (1.8%)	1 (0.9%)	3 (2.8%)	5 (4.7%)	1 (0.9%)	52 (49.0%)	106 ≈ (100%)

Abbreviations: AS, ankylosing spondylitis; BD, Behçet's disease; JRA, juvenile rheumatoid arthritis, OS, other spondylopathies; RV, retinal vasculitis; VKH, Vogt–Koyanagi–Harada disease.

While we assessed skin and mucous involvement, we found that five patients had two separate skin and mucous involvements, and two patients had three different involvements; 76 patients did not report any skin and mucous involvement; aphthous ulcer was the most common involvement, followed by nonsexually acquired genital ulcers (NSGU) and pseudo‐folliculitis barbae (PB). One hundred (94.33%) patients had no pulmonary complications, and only six (5.66%) had lung involvement. While approximately 66% of the patients had no joint involvement, among those with joint involvement, inflammatory back pain was the most common complication affecting 23 (21.7%) patients, followed by arthralgia affecting 12 (11.3%) patients and arthritis affecting 5 (4.7%) patients, two patients suffered from inflammatory back pain and arthritis simultaneously, and one patient was identified having inflammatory back pain, arthritis, and arthralgia at the same time.

**Figure 1:**
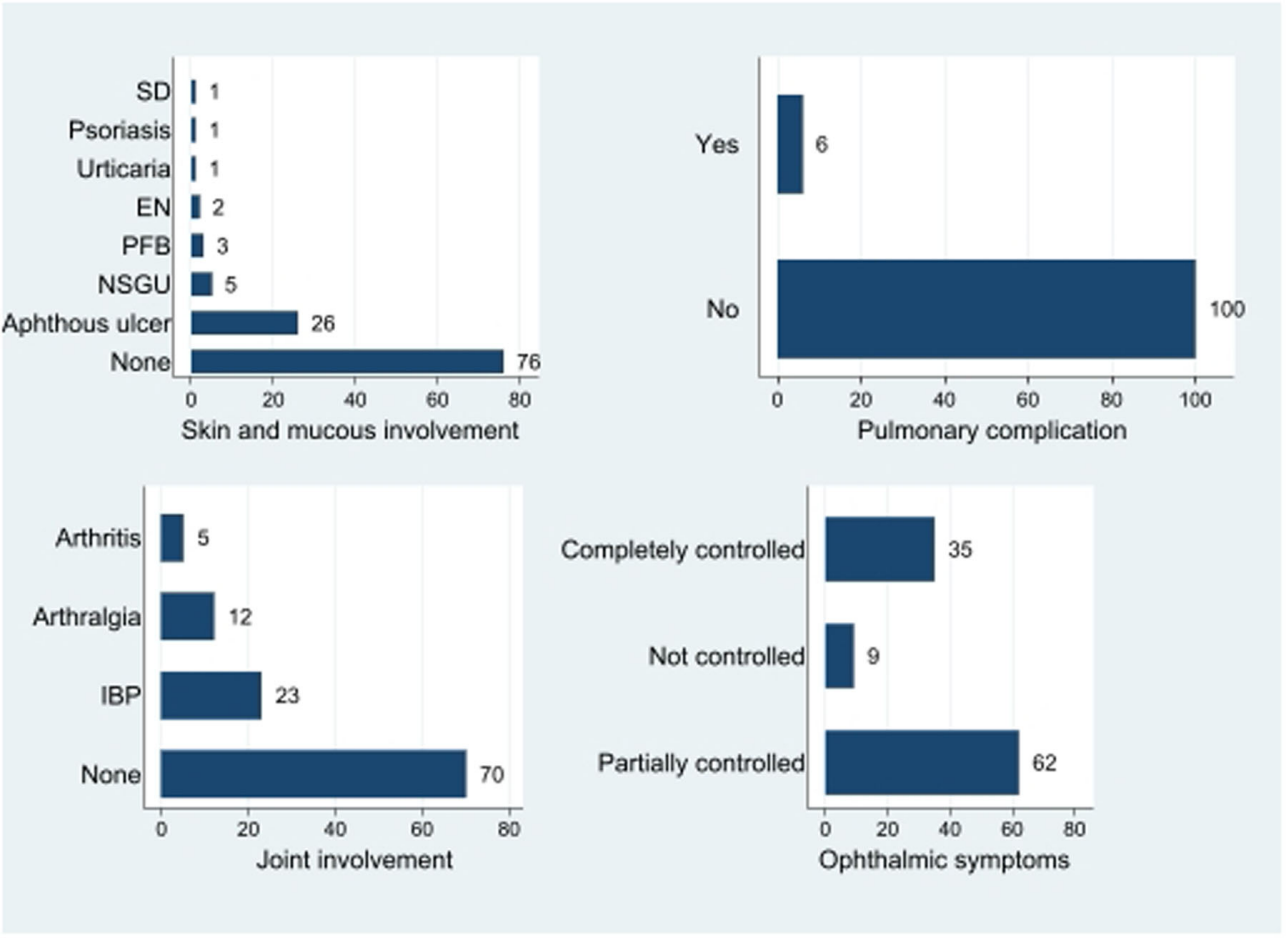
Pulmonary, joint, skin and mucous complications and follow‐up in patients. EN, erythema nodosum; IBP, inflammatory back pain; NSGU, nonsexually acquired genital ulcers; PFB, *Pseudofolliculitis barbae*; SD, seborrheic dermatitis.

Patients with regard to their ophthalmic symptoms were also followed up, and three different outcomes were classified: completely controlled, partially controlled, and not controlled; if the ophthalmic symptoms disappeared and the physical examination showed no abnormality, the patient's ophthalmic symptoms were considered being completely controlled; if the symptoms were relieved but the physical examination was abnormal, the patient's ophthalmic symptoms were considered being partially controlled, and if neither the symptoms were relieved nor the physical examination was normal, the patient's ophthalmic symptoms were considered not being controlled. Symptoms of 35 (33%) patients were completely controlled, 9 (8.5%) were not controlled at all, and 62 (58.5%) were controlled partially. The findings mentioned earlier are shown visually in Figure 1.

**Figure 2:**
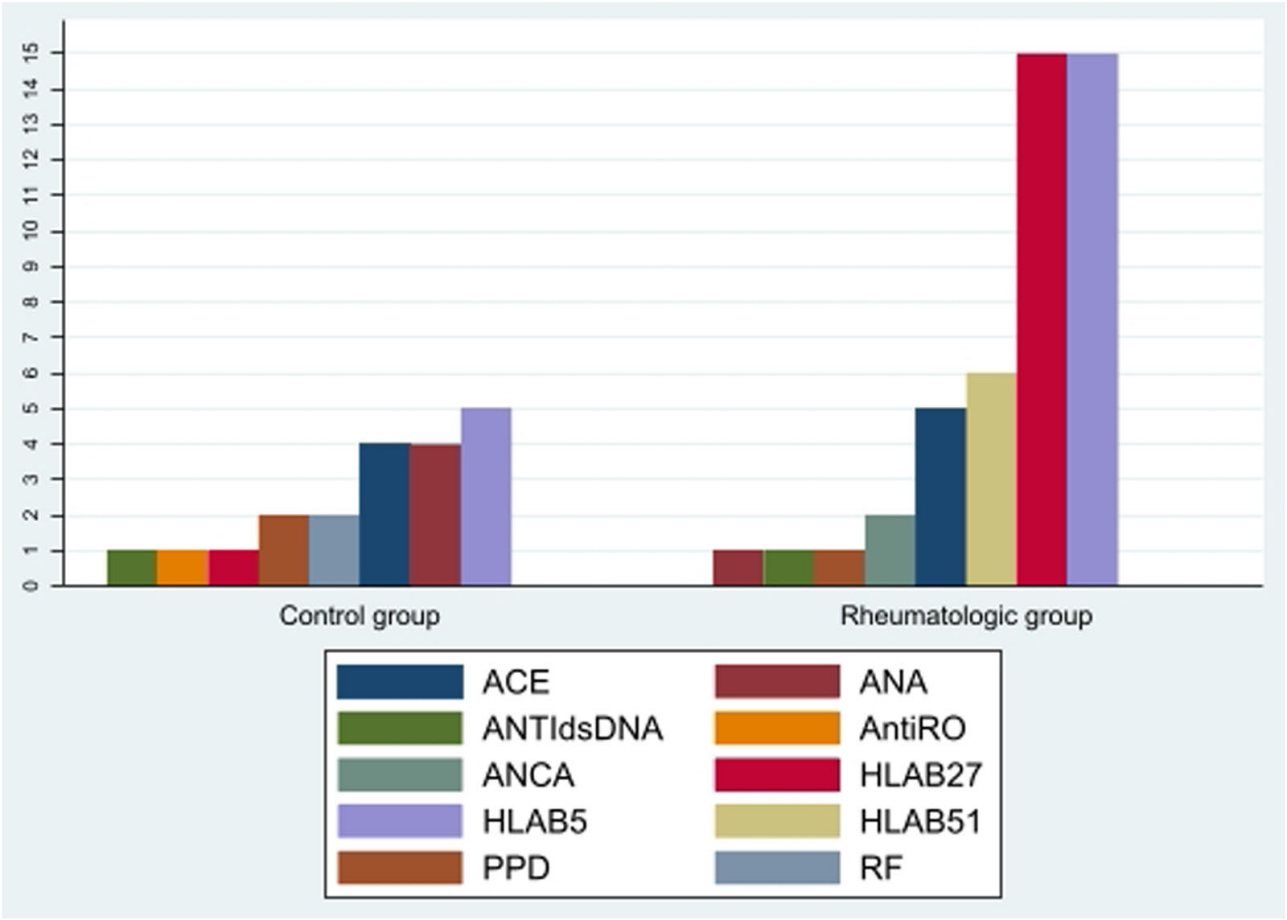
Lab tests in rheumatologic and control group. ACE, angiotensin‐converting enzyme; ANA, antinuclear antibody; ANCA, antineutrophil cytoplasmic antibody; PPD, purified protein derivative; RF, rheumatoid factor.

Patients in the rheumatologic disease group had more positive or elevated tests; the number of positive or elevated results for each test in this group were as follows: HLAB5 = 15, HLAB27 = 15, HLAB51 = 6, ACE = 5, ANCA = 2, ANA = 1, PPD = 1, ANTIdsDANA = 1, in addition, the number of positive or elevated results for each test in the control group were as follows: HLAB5 = 5, ANA = 4, ACE = 4, RF = 2, PPD = 2, HLAB27 = 1, AntiRO = 1, ANTIdsDNA = 1; Figure 2 visualizes laboratory test results.

Ophthalmic diseases were statistically associated with rheumatologic background (*p*‐value = .036), indicating that patients with rheumatic diseases more often had ophthalmic diseases than the control group; pulmonary (*p*‐value = .013), skin and mucous (*p*‐value < .001), and joint involvement (*p*‐value < .001) were also statistically different between two groups, suggesting patients with rheumatologic diseases (rheumatologic group) more frequently had pulmonary, skin, mucous, and joint involvement. Chi‐squared test results are listed in Table 4.

**Table 4:** Chi‐squared test results.

Test variables	Degrees of freedom	*χ* ^2^	*p*‐value
Association between ophthalmic diseases and having rheumatologic diseases	91	116.63	.036
Association between ophthalmic symptoms and having rheumatologic diseases	6	4.63	.592
Association between the sex of participants and their relative group	1	2.96	.085
Association between the age of participants and their relative group	42	52.56	.127
Association between the affected eye of participants and their relative group	2	3.37	.185
Association between the joint involvement of participants and their relative group	3	20.73	<.001
Association between the skin and mucous involvement of participants and their relative group	7	28.03	<.001
Association between the lung involvement of participants and their relative group	1	6.12	.013
Association between the outcome of patients' ophthalmic symptoms and their relative group	2	0.16	.920
Association between the positive or elevated lab tests of participants and their relative group	9	23.97	.004
Association between the comorbidities of patients and their relative group	9	12.27	.198

## 4 DISCUSSION

Based on the best of our knowledge, we disclose the first study, which presents ocular manifestations in patients with immune‐mediated rheumatologic diseases, and suspected AD patients in the Middle East and North Africa region. In our study, we included 106 patients with ophthalmic complications who were referred to rheumatologic clinics for further investigation.

Rheumatologic diseases have broad extra‐articular manifestations; skin, bones, eyes, mouth, and lungs can be affected frequently. On the one hand, a sophisticated ocular immune system and the blood‐aqueous barrier protect the eyes; not having a lymphatic outflow adds up to this protection. Still, on the other hand, they are commonly affected in rheumatologic diseases; not only early diagnosis of ocular involvement in rheumatologic diseases can prevent severe ophthalmic complications like vision loss and blindness, but also it can be a sensitive indicator for the severity of such diseases, having said that, familiarity with ophthalmic complications in rheumatologic diseases is essential for health care workers and especially for rheumatologists.^14^

Based on other studies, autoimmune diseases are far more common among females; similarly, in our study, most of the patients were also females (67%), and gender was not associated with immune‐mediated rheumatologic diseases in our study (*p*‐value = .085); this event might be because of our small sample size.^15^, ^16^, ^17^ We found that the mean age was 40.43 ± 13.26 years in our participants; Adelowo et al. and Pathanapitoon et al. reported the age of the participants 44.3 ± 13.7 and 48.9 ± 19.3 years, respectively, which were similar to our study; still, Uribe‐Reina et al. have reported the mean age 54.6 ± 15.6 years, which was higher than our results.^15^, ^18^, ^19^

Although approximately half of our participants were older than 40, only 16 patients reported comorbidities, and the comorbidities were not statistically associated with rheumatologic disease history (*p*‐value = .198). Involvement of a singular eye was more common (56.6%) than involvement of both eyes (43.40%), but the involvement of both eyes was more common than the isolated left or right eye involvement. The distribution of the affected eye was not significantly different in the control and rheumatologic group (*p*‐value = .185).

Blurred vision and eye redness were the most prevalent, and metamorphopsia and photophobia were the least frequent ophthalmic symptoms among patients. Ophthalmic symptoms were not associated with having rheumatologic diseases (*p*‐value = .592). In the Uribe‐Reina et al.,^15^ study, 35% of patients had at least one ocular symptom; the most frequent ones were dry eyes (30.8%), and ocular pain (2.7%), which is in contrast with our findings, yet the overall percentage of ocular pain in their study is similar to ours (1.8%); similarly, Ausayakhun et al.^20^ reported dry eyes (19.9%) as their most frequent ophthalmic symptom. Isolated anterior uveitis was the most observed ophthalmic disease, with 35.8% of total cases, which adds up to 41.2% of total cases if we consider anterior uveitis with or without other ophthalmic complications. Following anterior uveitis, posterior uveitis was the second utmost ophthalmic complication with an incidence of 20.7%, and panuveitis (17.9%) was the third most observed ophthalmic disease. In a study by Jiménez‐Balderas et al. on 57 patients diagnosed with uveitis, anterior uveitis was the most frequent type of uveitis, and vision loss was associated with uveitis recurrence.^21^ In a study conducted by Cakan et al.,^22^ the most frequent type of uveitis was anterior uveitis, followed by bilateral intermediate uveitis and bilateral pan uveitis.

A significant association exists between having a rheumatologic disease and having ophthalmic diseases (*p*‐value = .036). We diagnosed immune‐mediated rheumatologic diseases by order of frequency as follows: Behçet's Disease (21%), ankylosing spondylitis (17.9%), sarcoidosis (4.7%), other spondylopathies (2.8%), Vogt–Koyanagi–Harada disease (1.9%), juvenile rheumatoid arthritis, and isolated retinal vasculitis (0.9%). Other studies have reported different rheumatologic diseases' occurrence frequencies; in Tseng et al.^7^ study, they reported the frequency of rheumatologic diseases occurrence from more to less as follows: ankylosing spondylitis, Behçet's Disease, sarcoidosis, psoriasis, and juvenile rheumatoid arthritis; Uribe‐Reina et al.^15^ reported prevalence of rheumatologic diseases in their study population as follows: rheumatoid arthritis (33.3%), fibromyalgia (22.7%), Sjögren's syndrome (19.7%), and lupus (9.9%); and in Jiménez‐Balderas et al.^21^ study, ankylosing spondylitis was the most common rheumatologic disease.

We found that lung involvement was associated with rheumatologic background (*p*‐value = .013), and five patients out of six having lung involvement were diagnosed with sarcoidosis. Patients with rheumatologic diseases commonly had more positive and elevated laboratory tests, and laboratory tests were associated with rheumatologic background (*p*‐value = .004). Results on the outcome of ophthalmic symptoms were not associated with rheumatologic disease history, meaning patients with rheumatologic diseases necessarily did not have the worse ophthalmic symptoms outcome (*p*‐value = .920).

Investigating the frequency and outcome of ocular diseases among patients with autoimmune diseases is barely studied; to find more concerning ophthalmic symptoms with regard to diagnosing rheumatological diseases and to determine the most common ophthalmic symptoms, diseases, and other characteristics, including age, sex, the affected eye, comorbidities, laboratory tests, and other tissues involvement among patients with autoimmune diseases and suspected patients, this study was conducted. To better understand the key characteristics of patients with ophthalmic complications who develop rheumatologic diseases, we recommend further studies with larger sample sizes be conducted.

## 5 CONCLUSION

Since eye complications can be the first indicator of an autoimmune disease, understanding the prevalence of ocular symptoms and diseases among patients with autoimmune diseases is essential for proper referral to rheumatology clinics. Alternatively, early diagnosis of autoimmune diseases can prevent disastrous ophthalmic complications and improve patients' quality of life.

## 6 LIMITATION

The access to patients was limited due to the low prevalence of the disease, and the coronavirus disease restrictions further bounded our access to patients. To address this issue, we used medical records in rheumatology clinics.

## AUTHOR CONTRIBUTIONS

**Meharan Pournazari**: Conceptualization; data curation; methodology; project administration; resources; supervision. **Tara Hashemi**: Conceptualization; data curation; resources; validation. **Mahsa Zarpoosh**: writing—original draft; editing; Conceptualization; resources. **Parsa Amirian**: Formal analysis; project administration; visualization; writing—original draft; writing—review & editing.

## CONFLICTS OF INTEREST STATEMENT

The authors declare no conflicts of interest.

## Supporting information

Supporting information.

## Data Availability

The data that support the findings of this study are available from the corresponding author, upon reasonable request. The data sets used and analyzed during the current study are available by the corresponding author upon reasonable request.
